# Impact of mannitol on intracranial pressure assessed by optic nerve sheath ultrasonography during video-laparoscopic prostatectomy: a randomized clinical trial

**DOI:** 10.1016/j.bjane.2026.844733

**Published:** 2026-01-25

**Authors:** George Pereira Barreto, Ygor Paulion Bezerra Pereira, Isabelle França Bezerra Machado, Elkanah Marinho de Araujo, Fernanda Cunha Soares, Rand Randall Martins, Paulo José de Medeiros, Wallace Andrino da Silva

**Affiliations:** a Hospital Universitário Onofre Lopes, Division of Anesthesiology, Natal, RN, Brazil; b Universidade Federal do Rio Grande do Norte, Natal, RN, Brazil; c Karolinska Institute, Department of Dental Medicine, Division of Orthodontics and Pediatric Dentistry, Stockholm, Sweden; d Universidade Federal do Rio Grande do Norte, Department of Pharmacy, Natal, RN, Brazil; e Universidade Federal do Rio Grande do Norte, Department of Surgery, Natal, RN, Brazil

**Keywords:** Intracranial Pressure, Mannitol, Optic nerve, Prostatectomy ultrasonography

## Abstract

**Introduction:**

Intracranial pressure can increase during video-laparoscopic prostatectomy due to the Trendelenburg position and pneumoperitoneum, potentially leading to complications. The Optic Nerve Sheath Diameter (ONSD) has emerged as a reliable, non-invasive method to assess ICP. Mannitol is commonly used to reduce ICP, but its intraoperative effects in this surgical setting remain unclear. This study aimed to evaluate the impact of mannitol administration on ICP, as assessed by ONSD.

**Methods:**

This single-center, randomized, parallel-group, non-blinded clinical trial, 1:1 allocation, included 48 patients undergoing video-laparoscopic prostatectomy at a tertiary hospital in Brazil. Participants were randomly assigned to either the Mannitol Group, receiving 0.5 g.kg^-1^ of intravenous mannitol after 120 minutes in Trendelenburg, or the Control Group, which did not receive mannitol. ONSD was measured using ultrasonography at four intraoperative time points (T1–T4). Additional variables analyzed included hemodynamic and respiratory parameters, surgery duration, and extubation time. Statistical analysis was conducted using a linear mixed-effects model.

**Results:**

ONSD increased in both groups between T1 and T3, followed by a reduction at T4. However, the decrease in ONSD in the Mannitol Group was not statistically significant compared to the Control Group. Regarding extubation, the mean extubation time was 24.04 ± 15.71 minutes in the Mannitol Group and 22.79 ± 15.37 minutes in the Control Group (p = 0.782).

**Conclusion:**

Mannitol administration during video-laparoscopic prostatectomy did not result in significant differences in ONSD trajectory or extubation time compared with the control group. At the dose and timing used, mannitol did not modify intraoperative surrogate measures of intracranial pressure.

## Introduction

Prostate Cancer (PC) is the most common malignant neoplasm in men, with an estimated 1.6 million cases and 366,000 deaths annually, making it the second leading cause of cancer-related mortality in men.^1^^,^^2^ There are various treatment options for PC, with radical video-laparoscopic prostatectomy being widely performed as a definitive treatment for localized disease. However, to establish an adequate surgical field, a pneumoperitoneum with dioxide of Carbon (CO_2_) insufflation and Trendelenburg positioning (35‒45°) is required. These specific conditions alter normal physiology, leading to increased intra-abdominal, intrathoracic, and intracranial pressure due to a reduction in cranial venous drainage. Additionally, CO_2_ is known to cause cerebral vasodilation, leading to increased cerebral blood volume and flow. These factors collectively result in a significant increase in Intracranial Pressure (ICP).^3^

Intracranial pressure monitoring has traditionally relied on neuroimaging or invasive methods. Ultrasonographic measurement of the Optic Nerve Sheath Diameter (ONSD) has nevertheless emerged as a simple and practical surrogate for ICP assessment. Although several studies have suggested that an ONSD of approximately 5 mm may correlate with ICP values above 20 mmHg, these thresholds are context-dependent and show considerable heterogeneity across populations and methodologies.^4^

Intraoperative ICP elevation may lead to delayed or inadequate emergence from anesthesia, manifesting as delirium or disorientation.^5^ However, postoperative neurological complications or visual dysfunction have not been observed.^6^^,^^7^

Among the various strategies available to reduce ICP during surgery, mannitol plays a significant role. It is an osmotic diuretic that is easy to administer and relatively safe, increasing cerebral blood flow, reducing cellular edema through its osmotic properties, and improving cerebral oxygenation and microcirculation.^3^

This study aims to analyze the author’s hypotheses regarding the effect of mannitol in attenuating the increase in optic nerve sheath diameter compared with the control group, as well as its impact on extubation time in patients undergoing video-laparoscopic prostatectomy.

## Methods

A randomized, controlled, non-blinded clinical trial was conducted to evaluate patients undergoing video-laparoscopic prostatectomy at a Brazilian tertiary university hospital between May 2023 and October 2024. The study protocol was submitted to and approved by the Ethics Committee of Hospital Universitário Onofre Lopes (protocol number CAAE 55520021.5.0000.5292) and was registered in the Brazilian Registry of Clinical Trials (ReBEC) under number 6.573.095 on September 3, 2022.

### Participants

Male patients aged 20 to 79 years with a diagnosis of prostate cancer and willing to participate after signing the informed consent form were included in the study. Exclusion criteria comprised conditions that increase intracranial pressure, a history of neurosurgery, glaucoma, cardiac, hepatic, or renal insufficiency, an ASA classification greater than III, conversion of the surgery to an open modality, hemodynamic instability during surgery, and a surgical duration of less than 120 minutes.

### Randomization and intervention

Simple randomization with a 1:1 allocation ratio was performed using sequentially numbered, opaque, sealed envelopes prepared by a researcher not involved in patient enrollment. After anesthesia induction, the anesthesiologist opened the envelope and allocated each patient to one of two groups: the Mannitol Group (MG) or the Control Group (CG).

Patients in the MG received intravenous mannitol after 120 minutes in the Trendelenburg position, at a dose of 0.5 g.kg^-1^ infused over 30 minutes. Total body weight was used for dose calculation, with a maximum dose of 50 g. Patients in the CG did not receive mannitol, or any other intervention intended to reduce intracranial pressure. No changes to the study protocol or outcomes occurred after trial initiation. The timing of mannitol administration was standardized based on institutional workflow characteristics. As this is a teaching hospital where video-laparoscopic prostatectomies typically last 5 to 6 hours, and considering that approximately one-hour elapses between anesthetic induction and the beginning of the procedure itself, the authors selected this timing to ensure that mannitol would exert its osmotic effect during a substantial portion of the prolonged Trendelenburg period. This decision was supported by using the maximum dose of 50 g and the drug’s half-life of approximately 90–120 minutes.

All ONSD measurements were performed by staff anesthesiologists with more than five years of clinical experience and specific training in optic nerve ultrasonography. The anesthesiologist performing the measurements was not involved in the anesthetic management of the case and remained blinded to group allocation. Blinding was ensured because, at the times when ONSD measurements were taken, mannitol infusion in the MG either had not yet begun or had already been completed. Nevertheless, the study is considered unblinded overall, as other team members were aware of group assignment, given that a placebo infusion for the control group was not feasible.

ONSD was measured once in each eye at each of the four predefined intraoperative time points (T1–T4), and the mean value of both eyes was used for statistical analysis. According to the protocol, measurements would be repeated if the inter-eye difference at the same time point exceeded 0.5 mm; however, this threshold was never reached, and no repeated measurements were required. Using a GE HealthCare Logiq® ultrasound system with a 7.5 MHz high-frequency linear probe, ONSD was assessed 3 mm posterior to the globe at the following time points: T1 (10 minutes after induction), T2 (Trendelenburg position with 15 mmHg pneumoperitoneum), T3 (immediately before returning to horizontal position at the end of surgery), and T4 (10 minutes after resuming the supine position and pneumoperitoneum deflation). All measurements were performed by trained anesthesiology staff experienced in optic nerve ultrasonography.

The strategy was designed to minimize the risk associated with excessive ocular globe compression during surgery. Considering that each patient underwent four measurement time points per eye, performing three repeated measurements per eye at every time point would have resulted in 12 measurements per eye throughout the procedure, significantly increasing examination duration and potential risks without adding meaningful clinical benefit.

### Anesthetic management, data collection and outcomes

Patients underwent balanced general anesthesia, and the anesthesiologist determined whether neuraxial anesthesia would also be used. Pre-oxygenation was performed using a face mask at 8 L.min^-1^. Following orotracheal intubation, patients were ventilated in controlled mode with a tidal volume of 6–8 mL.kg^-1^, a Positive End-Expiratory Pressure (PEEP) of 5 cm H_2_O, and an inspired oxygen fraction of 50%. Depth of anesthesia is routinely monitored in our service. All patients were monitored with processed EEG (BIS®), and values were maintained between 40 and 60 throughout the procedure.

The primary outcome was the effect of mannitol administration on the trajectory of Optic Nerve Sheath Diameter (ONSD), measured in millimeters, across four intraoperative time points (T1–T4). Secondary outcomes included time to extubation, intraoperative hemodynamic parameters (e.g., mean arterial pressure), respiratory parameters (e.g., EtCO_2_ and peak inspiratory pressure), surgical duration, and intraoperative fluid volume.

### Statistical analysis

Sample size was calculated using Stata version 15 (StataCorp, College Station, TX, USA) to detect a mean difference of 0.3 mm in ONSD between groups, assuming a standard deviation of 0.3 mm, 80% statistical power, and a two-sided alpha of 0.05. Allowing for an anticipated 20% attrition rate, the final sample size was set at 24 patients per group. No missing data were observed for the outcomes evaluated.

The Minimal Clinically Important Difference (MCID) was defined as 0.3 mm based on prior perioperative ONSD studies showing that intraoperative changes during pneumoperitoneum and Trendelenburg typically range from 0.3 to 0.5 mm, and that mannitol-related effects fall within a similar magnitude.^3^ Additionally, studies correlating ONSD with invasively measured intracranial pressure indicate that differences of this magnitude may reflect clinically relevant changes around the ICP threshold of 20 mmHg.^4^ Published data also show intra- and inter-observer variability of approximately ± 0.1 to 0.3 mm, suggesting that a 0.3 mm difference exceeds expected measurement noise and likely represents a true physiological change. Therefore, a 0.3 mm difference was considered the smallest clinically meaningful effect of mannitol in this setting.^2^, ^3^, ^4^

Baseline characteristics were described using means and standard deviations for continuous variables and frequency distributions for categorical variables. Data normality was assessed using the Shapiro-Wilk test. Pre-intervention differences between groups were evaluated using Student’s *t*-test or Wilcoxon test for continuous variables and Pearson’s Chi-Square test or Fisher’s exact test for categorical variables. In addition, between-group differences in ONSD variation at each time point were analyzed using Student’s *t*-test, with a significance level set at p < 0.05.

A linear mixed-effects model was used to compare changes in ONSD between the Mannitol and Control groups. ONSD was considered the dependent variable. Time points (T1, T2, T3, and T4), group assignment, and their interaction were included as fixed effects. The model was additionally adjusted for Body Mass Index (BMI), surgery duration, and extubation time. Intra-individual variability was incorporated as a random effect to account for repeated measurements within subjects. The covariance structure for repeated measures was modelled as autoregressive of order 1 [AR(1)], assuming that measurements closer in time are more strongly correlated. The model included a random intercept for each subject to account for intra-individual variability across time points; random slopes were not included. Fixed effects comprised group, time, and group vs. time interaction, along with BMI, surgery duration, and extubation time as covariates. A p-value < 0.05 was considered statistically significant. Sample size calculation and statistical analyses were performed using Stata V 15 software. There were no missing data for the outcomes assessed. No subgroup or sensitivity analyses were performed.

## Results

**Figure 1:**
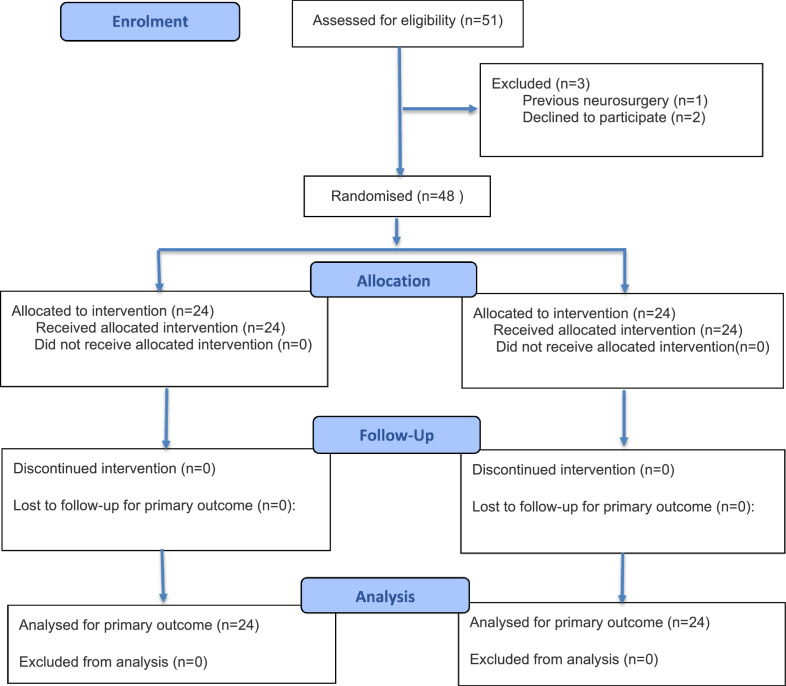
CONSORT flow diagram.

The study included 51 patients, with 48 patients randomized between the Mannitol Group (n = 24) and the Control Group (n = 24). None of them were excluded after randomization, as shown in Figure 1. All patients allocated to the mannitol group received the full dose as described, and no patient in the control group received mannitol.

No significant differences were observed in baseline characteristics, including age (p = 0.830) and BMI (p = 0.085), as demonstrated in Table 1.

**Table 1:** Demographic characteristics and results.

Variable	Mannitol Group	Control Group	Difference between means (95% CI)	p-value
Age (years)	64.71 ± 7.13	64.33 ± 4.63	−0.38 (−3.93 – 3.18)	0.83
BMI (kg.m^-2^)	24.95 ± 3.60	26.58 ± 2.76	1.63 (−0.12 – 3.38)	0.08
Surgery duration (hours)	6.51 ± 1.27	5.98 ± 1.30	−0.52 (−1.14 – 0.10)	0.16
Extubation time (minutes)	24.04 ± 15.71	22.79 ± 15.37	−1.25 (−10.56 – 8.06)	0.78
Infused volume (mL)	2161.78 ± 655.04	2120.03 ± 584.87	−41.67 (−265.08 – 181.175)	0.81
ETCO_2_ (mmHg) ‒ T1	32.63 ± 4.47	33.33 ± 8.06	0.71 (−2.57 – 3.99)	0.70
ETCO_2_ (mmHg) ‒ T2	34.88 ± 5.01	36.42 ± 6.12	1.54 (−1.99 – 5.08)	0.34
ETCO_2_ (mmHg) ‒ T3	37.88 ± 5.09	37.75 ± 3.91	−0.13 (−2.52 – 2.27)	0.92
ETCO_2_ (mmHg) ‒ T4	35.42 ± 5.19	35.92 ± 4.13	0.50 (−2.38 – 3.38)	0,71
MAP (mmHg) – T1	70.58 ± 14.27	69.33 ± 11.52	−1.25 (−9.39 – 6.89)	0,74
MAP (mmHg) ‒ T2	87.29 ± 16.78	88.08 ± 15.58	0.79 (−10.03 – 11.61)	0.87
MAP (mmHg) ‒ T3	76.54 ± 9,91	76.17 ± 10.56	−0.38 (−6.88 – 6.13)	0.90
MAP (mmHg) ‒ T4	72.21 ± 7.72	73.75 ± 10.74	1.54 (−3.16 – 6.25)	0.57
Peak inspiratory pressure (cmH_2_O) ‒ T1	18.58 ± 4.28	19.25 ± 5.70	0.66 (−2.53 – 3.87)	0.65
Peak inspiratory pressure (cmH_2_O) ‒ T2	27.00 ± 3.63	26.92 ± 5.68	−0.08 (−2.57 – 2.41)	0.95
Peak inspiratory pressure (cmH_2_O) ‒ T3	25.46 ± 5.62	24.96 ± 6.1	−0.50 (−3.62 – 2.62)	0.77
Peak inspiratory pressure (cmH_2_O) ‒ T4	19.67 ± 3.94	19.79 ± 3.61	0.13 (−1.93 – 2.18)	0.90
Mean eyes diameters (mm) ‒ T1	3.90 ± 0.56	3.57 ± 0.7	−0.33 (−0.73 – 0.08)	0.08
Mean eyes diameters (mm) ‒ T2	4.06 ± 0.93	3.93 ± 0.8	−0.13 (−0.67 – 0.40)	0.60
Mean eyes diameters (mm) ‒ T3	4.34 ± 0.85	4.39 ± 0.78	0.05 (−0.36 – 0.47)	0.83
Mean eyes diameters (mm) ‒ T4	3.95 ± 0.75	4.34 ± 0.95	0.39 (−0.04 – 0.83)	0.12

BMI, Body Mass Index; ETCO_2_, End-tidal Carbon Dioxide; MAP, Mean Arterial Pressure.

The analysis of intraoperative parameters showed that mean arterial pressure and end-ETCO_2_ levels were similar between groups at all time points (p > 0.05). The duration of surgery was slightly longer in the Mannitol Group (6.51 ± 1.27 hours) compared to the Control Group (5.98 ± 1.30 hours), but the difference was not statistically significant (p = 0.163). The volume of crystalloid infused during surgery also did not differ between the groups. No significant adverse events were recorded in either group.

Regarding extubation, the mean extubation time was 24.04 ± 15.71 minutes in the Mannitol Group and 22.79 ± 15.37 minutes in the Control Group (p = 0.782). The relative extubation time as a percentage of total surgical duration also did not differ significantly between groups (p = 0.650).

**Figure 2:**
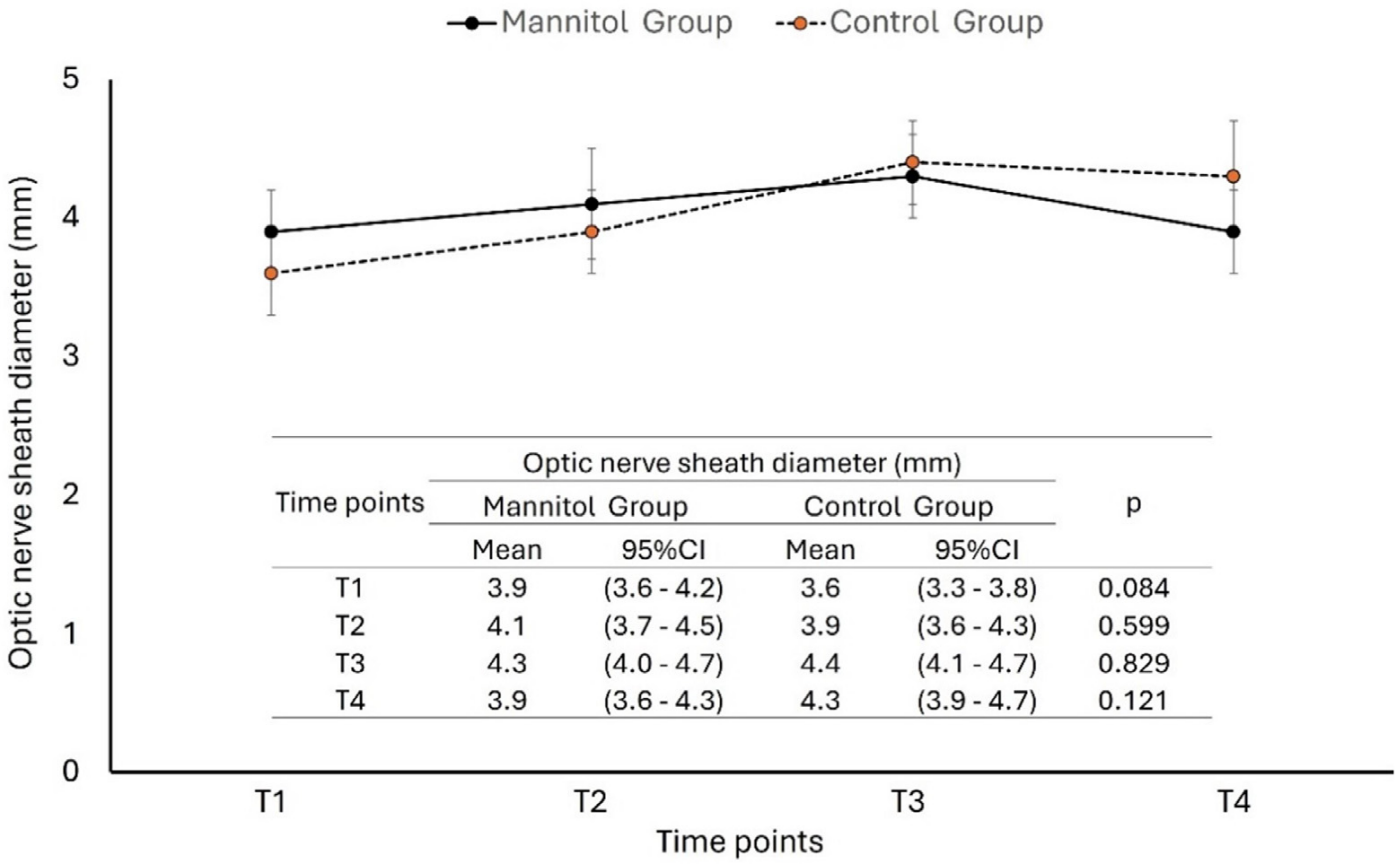
**Variation in Optic Nerve Sheath Diameter (ONSD) at four intraoperative time points (T1 to T4) in the Mannitol and Control groups.** Data are presented as mean values with 95% Confidence Intervals (95% CI). Between-group comparisons at each time point were performed using Student’s *t*-test; p < 0.05 were considered statistically significant.

Analysis of ONSD revealed a progressive increase from T1 to T3 in both groups, followed by a reduction at T4. The Mannitol Group showed a more pronounced decrease at T4 compared to the Control Group. However, between-group differences at each time point were not statistically significant, as shown in Figure 2. The figure displays mean ONSD values with 95% Confidence Intervals for each time point, along with results from univariate comparisons between groups.

In the mixed-effects linear regression model (Table 2), mannitol administration was not significantly associated with changes in ONSD (β = 0.206; 95% CI: -0.180 to 0.592; p = 0.984). None of the covariates included in the model ‒ BMI, surgery duration, or extubation time ‒ showed significant associations with ONSD variation. Only the intraoperative time points (T1, T2, T3 and T4) remained significantly associated with ONSD (β = 0.001; 95% CI: 0.000 to 0.002; p = 0.001).

**Table 2:** Mixed-effects linear regression model assessing the influence of mannitol on Optic Nerve Sheath Diameter (ONSD).

Optic nerve sheath diameter (mm)	Adjusted^a^		p
	β (SE)	95% CI	
Mannitol	0.206 (0.197)	−0.180 – 0.592	0.984
BMI (kg.m^-2^)	−0.024 (0.029)	−0.081 – 0.033	0.409
Surgery duration (hours)	−0.069 (0.076)	−0.219 – 0.080	0.364
Extubation time (minutes)	0.005 (0.006)	−0.007 – 0.018	0.389
Intraoperative time points (T1, T2, T3 and T4 in minutes)	0.001 (0.001)	0.000 – 0.002	**0.001**

a The dependent variable was the variation in optic nerve sheath diameter (ONSD, in mm). Fixed-effect independent variables included mannitol use, Body Mass Index (BMI), surgery duration, extubation time, and intraoperative time points. Time was modeled as a continuous fixed-effect variable. Intra-individual variability was included as a random effect to account for repeated measures within subjects. Estimates (β), Standard Errors (SE), 95% Confidence Intervals (95% CI), and p-values are reported.

## Discussion

The main findings of our study indicate that the Trendelenburg position is associated with an increase in the ONSD during video-laparoscopic prostatectomy. However, the administration of mannitol did not significantly alter the variation in ONSD, nor did it affect extubation time.

Several studies have demonstrated the use of ONSD measurement as an indirect indicator of ICP in surgeries requiring pneumoperitoneum and prolonged Trendelenburg positioning, such as conventional and robotic-assisted video-laparoscopic prostatectomies. Kim and colleagues assessed the extent of ICP elevation caused by CO_2_ pneumoperitoneum combined with the Trendelenburg position in patients undergoing robot-assisted laparoscopic radical prostatectomy. ONSD measurements and cerebral oximetry were recorded at five different time points during surgery. Their study, which included 20 patients, revealed a significant 12.5% increase in ONSD compared to post-induction values. Among these patients, three showed ONSD values corresponding to an ICP greater than 20 mmHg.^6^

Chin and co-investigators evaluated the sonographic measurement of ONSD as a surrogate marker for ICP in 21 anesthetized patients in the Trendelenburg position. Their findings demonstrated that ONSD measured three minutes after positioning was significantly higher (p < 0.001) than the value recorded after anesthesia induction (5.1 ± 0.3 mm vs. 4.5 ± 0.4 mm). This effect was also observed when Trendelenburg positioning was combined with pneumoperitoneum (4.9 ± 0.4 mm vs. 4.5 ± 0.4 mm). However, the final ONSD measurement after desufflation of pneumoperitoneum was comparable to the post-induction value.^8^

Balkan and collaborators investigated the effects of Trendelenburg positioning (35°–45° tilt) and CO_2_ insufflation on ONSD and hemodynamic parameters in patients undergoing robot-assisted laparoscopic radical prostatectomy to assess potential correlations between these variables. A total of 34 patients were included. ONSD was measured using ultrasound at four time points: T1 (5 minutes after intubation in the supine position), T2 (30 minutes after CO_2_ insufflation), T3 (120 minutes in the Trendelenburg position), and T4 (after abdominal desufflation in the supine position). Systolic and diastolic blood pressure, heart rate, and end-tidal CO_2_ were also recorded. The mean ONSD values were 4.87 mm at T1, 5.21 mm at T2, 5.30 mm at T3, and 5.08 mm at T4. A statistically significant increase in ONSD was observed between T1 and T3, followed by a significant decrease at T4. Additionally, a positive correlation was identified between ONSD and diastolic blood pressure, whereas no significant correlations were found between ONSD and mean arterial pressure, heart rate, or end-tidal CO_2_.^5^

The potential of mannitol as an intraoperative ICP-lowering agent has been explored with mixed results. Jun et al. reported a reduction in ONSD following mannitol administration in patients positioned in Trendelenburg during robotic prostatectomy, suggesting a potential benefit in mitigating ICP elevation.^3^ However, their study lacked a control group and was not randomized, limiting the generalizability of the findings. In contrast, our controlled data suggest that mannitol administration does not confer additional benefit in reducing ONSD or improving immediate postoperative outcomes in this specific surgical setting.

It is important to note that mannitol's pharmacological effects may be more pronounced in settings of sustained or pathological ICP elevation, rather than the transient increases seen during laparoscopic procedures in otherwise healthy individuals. Moreover, factors such as baseline intracranial compliance, the duration of surgical positioning, and individual variation in cerebrovascular autoregulation may modulate the impact of osmotic therapy on ICP and its surrogates.

Our study has several limitations. First, the potential variability in ultrasonographic technique could introduce measurement bias, including the possibility of intra-observer variation despite standardized training. Second, we did not assess perioperative neurocognitive outcomes, such as postoperative delirium or cognitive dysfunction. Although initially considered, these evaluations were not feasible in our institutional setting, particularly because standardized preoperative assessments could not be performed. Third, the relatively small sample size may limit the detection of subtle differences in secondary outcomes and affect the overall precision of our estimates. Finally, because this was a single-center study conducted in a teaching hospital with specific workflow characteristics, the generalizability of our findings to other settings may be limited.

## Conclusion

In this randomized trial, intraoperative mannitol administration during video-laparoscopic prostatectomy did not produce statistically significant between-group differences in ONSD trajectory, hemodynamic or respiratory parameters, or extubation time. Under the specific conditions, dose, and timing used in this study, mannitol did not modify surrogate measures of intracranial pressure. These findings should be interpreted cautiously as their generalizability to other populations, surgical durations, and dosing strategies may be limited.

## Data availability statement

The datasets generated and/or analyzed during the current study are available from the corresponding author upon reasonable request.

## AI assistance disclosure

The authors declare that no Artificial Intelligence (AI) tools were used in the conception, design, data analysis, or writing of this manuscript.

## Brazilian Registry of Clinical Trials (ReBEC)

Number 6.573.095 on September 3, 2022.

## Ethics Committee approval

Ethical approval was granted by the Onofre Lopes University Hospital Research Ethics Committee, under the Ethical Appreciation Presentation Certificate number CAAE 55520021.5.0000.5292 on May 4, 2022.

## Authors' contributions

George Pereira Barreto contributed to the study conception, conducted the study execution, and participated in manuscript drafting. Fernanda Cunha Soares and Rand Randall Martins contributed to statistical analysis and manuscript drafting. Paulo José de Medeiros contributed to the critical revision of the manuscript. Ygor Paulion Bezerra Pereira, Isabelle França Bezerra Machado, and Elkanah Marinho de Araujo were involved in the execution of the study. Wallace Andrino da Silva led the study, developed the original idea, coordinated all phases of the project, and was responsible for writing and revising the manuscript.

## Funding

This research did not receive any specific grant from funding agencies in the public, commercial, or not-for-profit sectors.

## Conflicts of interest

The authors declare no conflicts of interest.
